# DeepD2V: A Novel Deep Learning-Based Framework for Predicting Transcription Factor Binding Sites from Combined DNA Sequence

**DOI:** 10.3390/ijms22115521

**Published:** 2021-05-24

**Authors:** Lei Deng, Hui Wu, Xuejun Liu, Hui Liu

**Affiliations:** 1 School of Computer Science and Engineering, Central South University, Changsha 410075, China; leideng@csu.edu.cn (L.D.); wuhui@csu.edu.cn (H.W.); 2 School of Computer Science and Technology, Nanjing Tech University, Nanjing 211816, China

**Keywords:** protein–DNA binding, convolutional neural network, Word2Vec, transcription factor binding sites, bidirectional long short term memory network

## Abstract

Predicting in vivo protein–DNA binding sites is a challenging but pressing task in a variety of fields like drug design and development. Most promoters contain a number of transcription factor (TF) binding sites, but only a small minority has been identified by biochemical experiments that are time-consuming and laborious. To tackle this challenge, many computational methods have been proposed to predict TF binding sites from DNA sequence. Although previous methods have achieved remarkable performance in the prediction of protein–DNA interactions, there is still considerable room for improvement. In this paper, we present a hybrid deep learning framework, termed DeepD2V, for transcription factor binding sites prediction. First, we construct the input matrix with an original DNA sequence and its three kinds of variant sequences, including its inverse, complementary, and complementary inverse sequence. A sliding window of size *k* with a specific stride is used to obtain its *k*-mer representation of input sequences. Next, we use word2vec to obtain a pre-trained *k*-mer word distributed representation model. Finally, the probability of protein–DNA binding is predicted by using the recurrent and convolutional neural network. The experiment results on 50 public ChIP-seq benchmark datasets demonstrate the superior performance and robustness of DeepD2V. Moreover, we verify that the performance of DeepD2V using word2vec-based *k*-mer distributed representation is better than one-hot encoding, and the integrated framework of both convolutional neural network (CNN) and bidirectional LSTM (bi-LSTM) outperforms CNN or the bi-LSTM model when used alone. The source code of DeepD2V is available at the github repository.

## 1. Introduction

Transcription factor (TF) is a type of protein that controls the activity of genes, through binding to the upstream regulatory elements located in the promoter and enhancer regions. TF binding site, often referred to as motif, is a short nucleotide fragment that can be bound by TFs and thus determines the specificity of a DNA sequence. Identification of transcription factor binding sites is a key step for us to understand the mechanism of transcriptional regulation [1,2].

During the past few decades, with the advancement of high-throughput sequencing technology, a few experimental methods, such as Chromatin Immunoprecipitation-sequence (ChIP-seq), have been developed to identify protein–DNA binding sites [3,4,5]. ChIP-seq promotes to reveal the sequence patterns and greatly increases the spatial resolution of protein–DNA interactions. Although the amount of available protein–DNA binding sites increases rapidly, the DNA sequences extracted directly from ChIP-seq are still noisy [6]. In addition, ChIP-seq assays often require a nontrivial amount of tissue biopsy that are often difficult to obtain. Therefore, computational methods have been developed to predict protein–DNA binding sites. These methods can be roughly classified into conventional [7,8,9,10,11] and deep-learning algorithms [12,13,14,15,16,17,18,19,20,21].

Deep learning have been widely used in the bioinformatics field. Convolutional neural networks (CNNs) [22] have recently gained substantial advancement in motif elucidation [23,24,25]. For instance, Babak et al. [12] first applied deep learning to protein–DNA binding prediction. They developed an algorithm called DeepBind that achieves scalable and efficient performance by virtue of its deep convolutional architecture. A recurrent neural network (RNN) is also used in discovery of TF binding sites. DanQ [14] developed a hybrid convolutional and recurrent neural network framework for predicting a non-coding function of DNA directly from a sequence alone. WSCNNLSTM [18] proposed that the *k*-mer encoding can significantly improve the performance of modeling in vivo protein–DNA binding. Moreover, the performance of WSCNN [17] and WSCNNLSTM [18] can be promoted with the value of k increasing. However, too big *k* will cause an exponential growth of the number of parameters that are computationally inhibitive.

Although previous methods based on deep learning are effective in extracting information from DNA sequences to predict protein–DNA bindings, these methods suffer from several limitations: (1) one-hot and *k*-mer encoding used for sequence feature extraction are vulnerable to high-dimensional problems. (2) The distance among sequences is close. For example, ATCCG differs more from GAGCA than ATCCC, but they have the same distance according to one-hot encoding [26]. This results in sparsity and less informative input of the deep learning model. (3) Previous methods often take as input only original DNA sequences, which often leads to the loss of latent information contained in its inverse, complementary, and complementary inverse sequences.

To overcome the limitations mentioned above, we proposed DeepD2V, a novel deep neural network model for the prediction of protein–DNA binding sites. Our method contributes to at least three novelties: (1) dna2vec is adopted to compute distributed representations of variable-length *k*-mer sequences. (2) We construct a combination input matrix from original, complementary, and inverse complementary DNA strands, and then mapped each *k*-mer sequence into a unified vector space. (3) We combined the CNN and RNN components into our end-to-end learning framework. In fact, DeepD2V is designed to learn motif features from input sequences through convolution filters, and then use bi-directional long short-term memory (bi-LSTM) to capture high-order structural features. The performance of DeepD2V was extensively evaluated on 50 public ChIP-seq benchmark datasets released by the HaibTfbs group, and the performance comparison results demonstrated that DeepD2V outperforms several popular methods for predicting protein–DNA binding. We believe that our method would significantly contribute to the prediction of protein–DNA binding sites and understanding of transcriptional regulation mechanism.

## 2. Materials and Methods

### 2.1. Data Source

We collected 50 public ChIP-seq data sets from the ENCODE project [27] to assess the performance of the proposed method DeepD2V. The data sets originate from three types of cell lines, including Gm12878, H1-hESC, and K562. For each cell line, we selected ∼15,000 top-ranking sequences as positive samples from each record in a peak file where each sequence consists of 200 base pairs.

Negative sequences are generated to match the statistical properties of the positive set [2,28,29]; otherwise, the generated data set may lead to biased [30] experiment results. The negative samples were generated by matching the repeat fraction, length, and GC content of the positive ones following the work of Ghandis et al. [28]. We generate three data sets with different ratios of negative to positive samples to evaluate the robustness of the proposed method. The ratio of negative to positive is 1:1, 2:1, and 3:1, respectively. It is worth noting that the ChIP-seq data and training set preparation have been adopted by DeepBind, DanQ, WSCNN, and WSCNNLSTM.

To evaluate the performance of DeepD2V, we adopted three-fold cross validation to tune the model parameters. The benchmark dataset was split into three folds randomly, two of which were used as a training set and the remaining as a test set. The process was repeated for three times and the average of performance metrics are computed. In addition, 1/8 training samples were randomly sampled as a validation set. The data set used in this paper can be freely accessed at http://pre3sdn.denglab.org/rawdata.zip (accessed on 30 April 2021).

### 2.2. Sequence Conversion and Representation

First, we derived three other variant sequences from an original DNA sequence, including the complementary, inverse, and complementary inverse sequences. The four sequences are concatenated in a specific order into the dna2vec method to derive the distributed representation matrix.

Afterward, the combined sequences were segmented according to the *k*-mer method, which is often used in sequence analysis. *K*-mer is used to divide a sequence into multiple substrings with *k* bases. With the stride size 1, a sequence with *l* bases is divided into (*l*−*k* + 1) *k*-mers. For example, the sequence AGCCT is split into three 3-mers: (AGC, GCC, CCT). Accordingly, the *l*-length DNA sequence is split into *k*-mer subsequences with *k* bases to form a new sequence with length ($\frac{\left(l-k\right)}{s}+1$) words in total.

**Figure 1:**
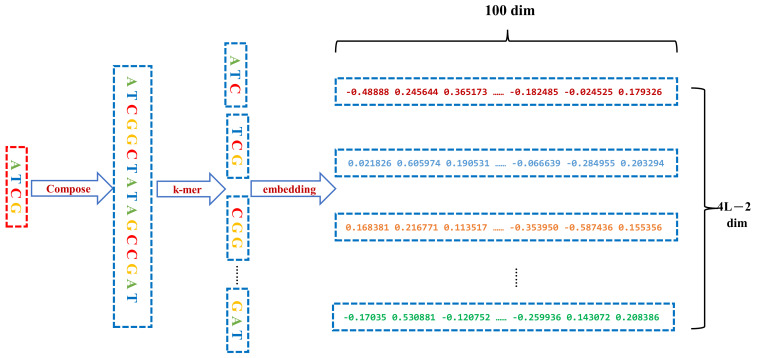
Each input DNA sequence of 200 bp concatenated by its three types of variant sequences so that a 800 bp sequence is constructed, which is followed by *k*-mers encoding and distributed representation by the word2vec algorithm.

We regard the entire DNA sequence as a sentence, and *k*-mer segments as the words that make up the sentence. Therefore, the word2vec model, a continuous bag-of-word Model (CBOW) model, is used to train all the processed sequence corpus to generate sequence word vectors. The CBOW model predicts the probability of the occurrence of the target word according to the context. Each target word is represented by a 100-dimensional vector. After several rounds of iterations, each original base sequence of length 200 bps was converted to a (798,100) matrix. The sequence conversion and representation are illustrated in Figure 1.

### 2.3. Model Architecture

**Figure 2:**
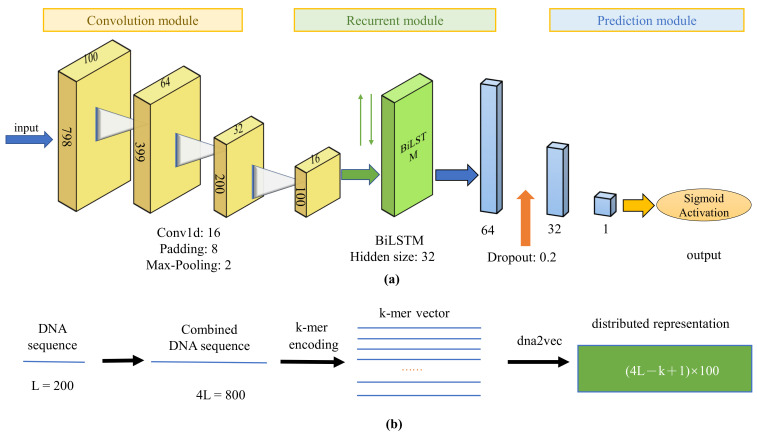
The illustrative diagram of our DeepD2V method for predicting protein–DNA binding. (**a**) The DeepD2V model includes three modules: convolution modules, recurrent module, and prediction module. Each convolutional module consists of four layers including convolution layer, rectified linear unit, batch normalization, and pooling; (**b**) DeepD2V takes as each input a DNA sequence of 200 bp, and generate three types of variant sequences concatenated to form a 800 bp sequence, followed by *k*-mers encoding and distributed representation by a dna2vec algorithm.

We first present the illustrative diagram of DeepD2V, as shown in Figure 2. The convolution module is used to extract features from input sequences, and then the Bi-LSTM module is used to capture high-order features, followed by two fully-connected layers and a dropout layer for prediction.

The function of each module is described in detail as follows. The entire workflow can be formulated as Equation (1):

(1) $$Y=f_{pred}\left(f_{rnn}\left(f_{cnn}\left(x\right)\right)\right)$$

Compared with an RNN, a one-dimensional convolutional neural network can shorten the sequence and extract the high-order feature of motifs, but requires a small computational cost when dealing with long biological sequences. Therefore, we use a one-dimensional convolution module before RNN. Each convolution module consists of a one-dimensional convolutional layer, a rectified linear layer (ReLU) [31], and a max pooling layer. A convolution layer is responsible for capturing motif features with a specified number of kernels of filter. Relu is used as an activation function because it can reduce the gradient descent and back propagation efficiently, avoiding gradient explosion and gradient disappearance. Finally, a max pooling layer is employed to reduce redundant information of the output of convolutional layer and choose the maximum response in the filters.

Long Short-Term Memory (LSTM) [32] is a variant of RNN, which solves the problem that traditional RNN cannot manage with long-term dependence. In consideration of the double-stranded structure of the DNA sequence, we selected bi-LSTM to extract long-term features of the sequence. The specific calculation formula of bi-LSTM unit at position *t* is presented as follows:

(2) $$\begin{array}{cc} \; & f_{t}=\sigma \left(W_{f}x_{t}+U_{f}h_{t-1}+b_{f}\right),\\ \; & i_{t}=\sigma \left(W_{i}x_{t}+U_{i}h_{t-1}+b_{i}\right),\\ \; & c_{t}=f_{t}⊙c_{t-1}+i_{t}⊙tanh\left(W_{c}x_{t}+U_{c}h_{t-1}+b_{c}\right),\\ \; & o_{t}=\sigma \left(W_{o}x_{t}+U_{o}h_{t-1}+b_{o}\right),\\ \; & h_{t}=o_{t}⊙tanh\left(c_{t}\right) \end{array}$$

where ⊙ is element-wise multiplication, *b*${}_{o}$, *b*${}_{c}$, *b*${}_{i}$, and *b*${}_{f}$ are the biases, and *W*${}_{i}$, *W*${}_{f}$, *W*${}_{o}$, *U*${}_{o}$, *U*${}_{f}$, and *U*${}_{i}$ are the weights.

Two fully connected layers and one dropout layer [33] constitute the final prediction module to integrate the feature learned from CNN and RNN. Dropout is widely used for regularization to avoid overfitting by reducing the complex co-adaptation relationship between neurons in a deep neural network. The dropout ratio is tuned via cross-validation. Finally, a sigmoid active function is used to compute the probability of protein–DNA binding.

### 2.4. Implementation and Hyperparameter Optimization

The DeepD2V model is implemented by PyTorch 1.0, and the source code and the data set is available at https://github.com/Sparkleiii/DeepD2V (accessed on 30 April 2021). Hyper-parameters were sampled randomly from search space on each ChIP-seq data set and adjusted to optimal values via cross-validation. We initialized all weights through Xavier uniform distribution [34] and initialized all biases to zero.

The DeepD2V model reaches convergence within 20 epochs, and the train–loss curve decrease gradually and become steady after 20 epochs. Thus, we set the training epoch to 20, and the model with the highest ROC AUC value on the validation set was selected as the final best model. The performance metrics of the best model on the test set was recorded, and the average of the three-fold cross-validation results was considered as the final result. Following the result of previous study [19], the number of convolution kernels was set to 16.

The initial value of learning rate is set to 0.001 and adjusted with the training epochs dynamically through an Adam [35] optimizer. A DeepD2V model was trained on the workstation with a Tesla M10 GPU, and training process on 50 data sets finished within seven hours. The number of sequences simultaneously considered in gradient calculation was determined by batch size. A large batch size can usually speed up the training process effectively, but an oversized batch size easily results in a local minimum. Therefore, the batch size in our study was set to the commonly used value 64.

Table 1 summarizes recommendations and starting points for the most common hyper-parameters.

## 3. Results

We first assessed the robustness of DeepD2V with different strides and *k*-mer values. To verify the performance improvement by combined DNA sequences, the performance of DeepD2V model with different inputs was compared. Finally, DeepD2V was compared to other simplified models with only CNN, RNN or Bi-LSTM modules alone.

For systematic performance evaluation, we compare DeepD2V with other four state-of-the-art protein–DNA binding methods, including DanQ, DeepBind, WSCNN, and WSCNNLSTM. The performance comparison experiments were conducted on 50 ChIP-seq benchmark data sets.

### 3.1. Parameter Optimization for k-Mers and Stride

Given the fact that we employed the dna2vec method to obtain distributed vector representations of each *k*-mer, the best values for *k* and stride *s* can be determined by experimentally comparing the performance of different combinations of *k* and *s* values on the 23 ChIP-seq data sets of a Gm12878 cell line.

**Figure 3:**
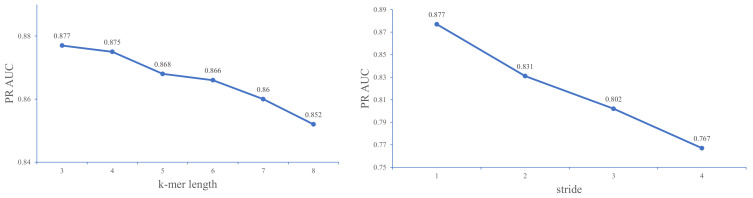
A comparison of DeepD2V with different *k*-mer length and stride step on the 23 ChIP-seq datasets in a Gm12878 cell line under the PR AUC metric.

As shown in Figure 3, when the value of *k* increases, the performance of the model gradually decreases. The model achieves better performance at *k* = 3. We suppose that this result may be attributed to the fact that three bases constitute an amino acid, and *k* = 3 exactly captures the biological essentiality of the sequence. For the subsequent tuning of stride step *s*, we set *k* to 3 and then use grid search for an optimal value of *s*.

Figure 3 shows that the larger the stride, the worse the performance of the model. This result may be due to the large stride leading to a loss of fine-grained sequence information. Previous studies [36] encourage the use of larger values of *k*-mer length and suggests that small stride values (*s* = 1) may decrease the performance of the embedding algorithm. However, in our empirical experiments, we find that the model with smaller values of stride and *k*-mer length (*k* = 3 and *s* = 1) perform better than other combinations of *k* and *s*. Therefore, we set the *k* and *s* values to 3 and 1 in the following performance comparison experiments. Note that the experiment utilizes a raw DNA sequence as input instead of the combined sequence.

### 3.2. Distributed Representation Significantly Improves the Prediction Performance

**Figure 4:**
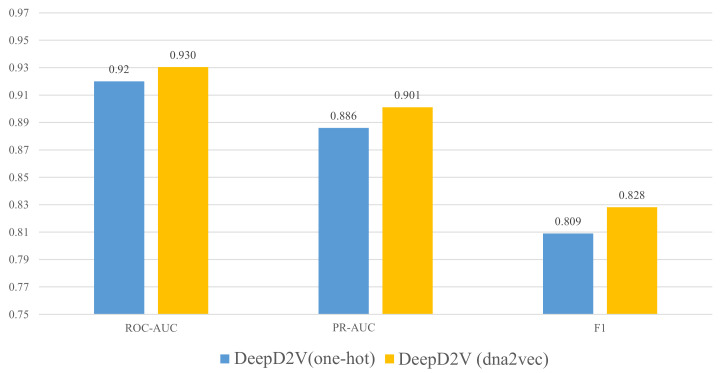
Performance comparison of DeepD2V using one-hot encoding vs. dna2vec on 50 in vivo datasets.

Rather than one-hot encoding of *k*-mers, our model adopts word2vec to construct *k*-mers distributed representation. Although word embedding has been widely used in the field of natural language processing [37], it is still seldomly applied in protein–DNA binding research [38]. To verify the advantage of dna2vec [26], we performed several comparative experiments on 50 public in vivo ChIP-seq benchmark datasets. Figure 4 illustrates the performance of DeepD2V under the average AUC and F1-score metrics using one-hot encoding and dna2vec. We can conclude that DeepD2V using dna2vec significantly outperforms one-hot encoding on all three metrics.

**Figure 5:**
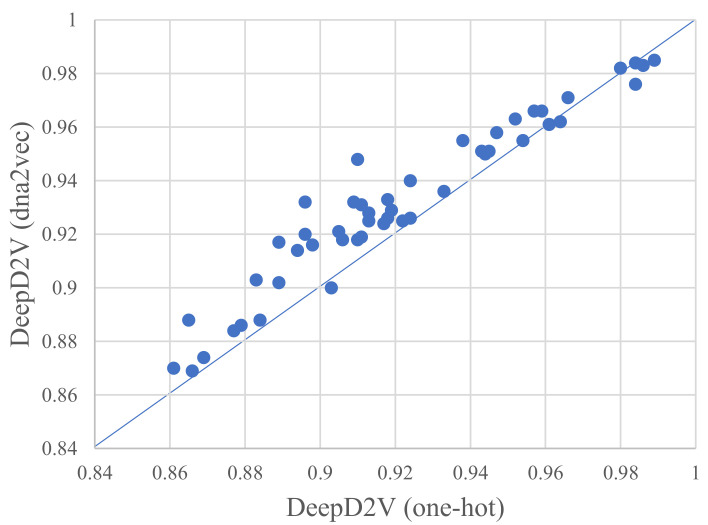
A comparison of DeepD2V uses one-hot encoding and DeepD2V use dna2vec on 50 public ChIP-seq datasets under the ROC AUC metric.

As shown in Figure 5, the ROC AUC measures between one-hot encoding and dna2vec on 50 public ChIP-seq datasets. Furthermore, DeepD2V performs better with dna2vec-derived distributed representation, again one-hot encoding. The reason may lie in the fact that a dna2vec embedding vector explicitly considers the distance and abstract dependencies among *k*-mers (including implicit DNA structure information). For detailed results of the experiments, please see the Supplementary File.

### 3.3. Combined Sequence Outperforms Original DNA Sequence

**Figure 6:**
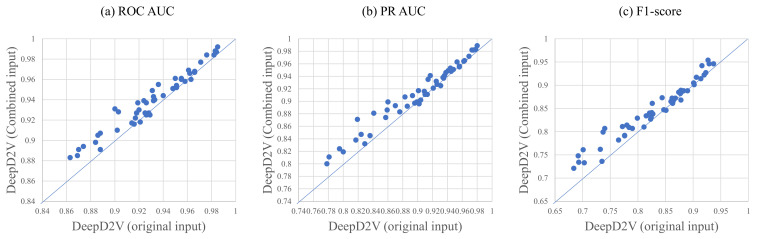
A comparison of DeepD2V with original input and DeepD2V with combined input on in vivo data, where the (**a**–**c**) is under the ROC AUC metric, PR AUC metric, and F1-score metric, respectively.

For double-stranded DNA sequences, reverse or complement sequences are taken into account in the training of DeepD2V because proteins may bind to the DNA complementary or reverse the sequence strand. Hence, we explore the significance of a combined sequence and the original DNA sequence again. Figure 6 shows the performance of DeepD2V taking as input the original DNA sequence and combined sequences on all 50 benchmark data sets. It can be found that the combined sequences greatly promote DeepD2V performance compared to original DNA sequences over all of the three performance measures. We think that the combined sequences potentially capture high-order dependencies among different patterns of DNA sequences, such as implicit DNA shape.

### 3.4. Performance Comparison between CNN, RNN, bi-LSTM, and DeepD2V

**Figure 7:**
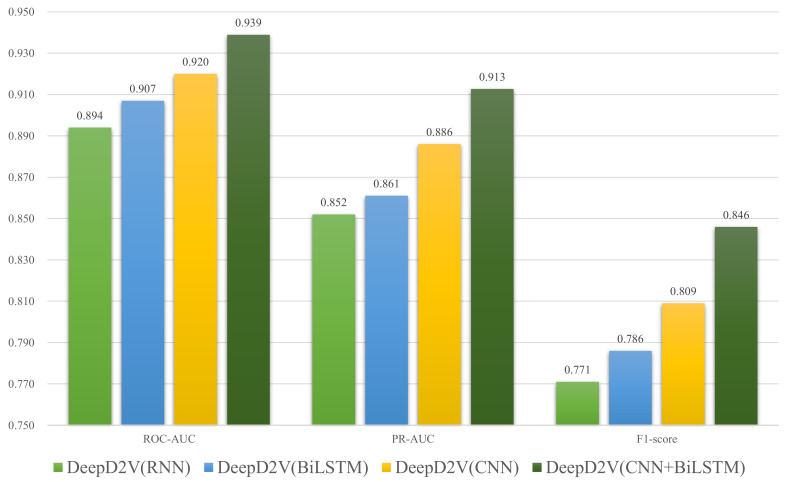
Comparison of DeepD2V with CNN-only, RNN-only, and hybrid structure of CNNs and RNNs on 50 public ChIP-seq datasets under the ROC AUC, PR AUC, and F1-score metrics.

To verify the performance improvement by the combination of CNN and bi-LSTM, we compare DeepD2V to three simplified models: CNN-only, RNN-only, and the hybrid structure of CNNs and RNNs. Figure 7 shows the performance of DeepD2V with these deep learning architectures on all 50 public ChIP-seq data sets. It should be noted that the specific hyper-parameters of the four models have been optimized so that objective performance evaluation can be done. We think CNN performs well in extracting high-order features from sequences, while bi-LSTM can capture the long-term dependence between motifs, and avoid the problem of vanishing gradient and exploding gradient in the process of DNA long sequence training. Compared with CNN or bi-LSTM alone, DeepD2V achieved better prediction performance with an average F1-score of 0.809. DeepD2V takes full advantage of the CNN and bi-LSTM model and thus achieves the best performance. It extracts the high-order abstract features from the combined DNA sequences by CNN and captures bidirectional semantic dependencies of sequences with bi-LSTM.

### 3.5. Performance Comparison with Competing Methods

To better evaluate the performance of DeepD2V, we compare DeepD2V with three deep-learning based models, including DeepBind, DanQ, and WSCNNLSTM, on 50 ChIP-seq benchmark datasets. All competing methods are run using the source code released by their authors. In addition, we optimize the hyper-parameters of these competing methods via cross-validation to make the model achieve the best performance on the test set. This makes the performance comparison to our method objective.

**Figure 8:**
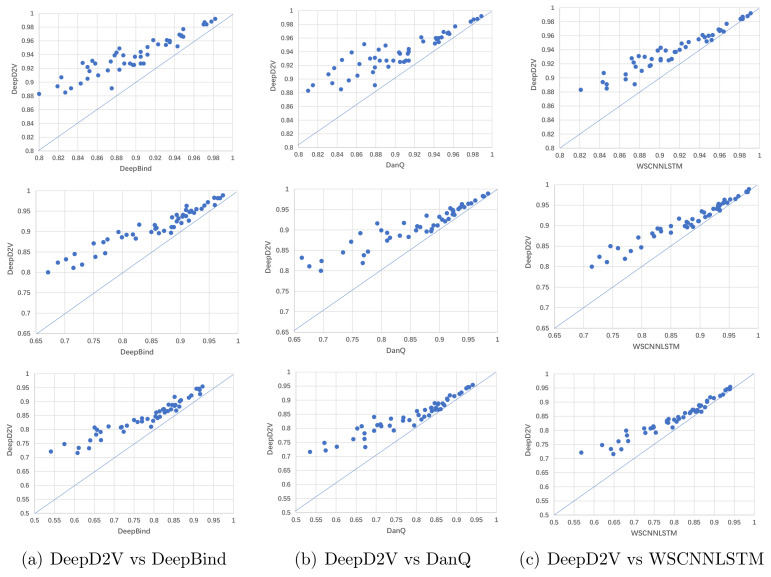
Comparison of DeepD2V and the competing methods on in vivo data. (**a**) Comparison of DeepD2V with DeepBind under the ROC AUC, PR AUC and F1 metrics. (**b**) Comparison of DeepD2V with DanQ under the ROC AUC, PR AUC and F1 metrics. (**c**) Comparison of WSCNNLSTM with DeepBind under the ROC AUC, PR AUC and F1 metrics.

The AUCs and F1-scores across 50 ChIP-seq datasets are shown in Figure 8. Each point in the scatter plot represents the performance measures of two methods on the same dataset. The point located on the top left of the diagonal shows that the performance of DeepD2V model is better than counterpart methods.

Figure 8a shows that the performance of DeepD2V was apparently higher than that of DeepBind in F1-score and AUC metrics. This advantage may be attributed to the combined input and added an RNN layer to capture the long-term dependence of motifs, which is not applied in DeepBind. In addition, DeepD2V significantly outperformed DanQ according to the F1-score and AUC metrics, as shown in Figure 8b. This result proves that the distributed dense representation of dna2vec outperforms one-hot encoding sparse representation. As shown in Figure 8c, DeepD2V performs better than WSCNNLSTM based on the F1-score and AUC metrics, demonstrating that the combined input can extract more effective motif features than the original DNA sequence.

**Figure 9:**
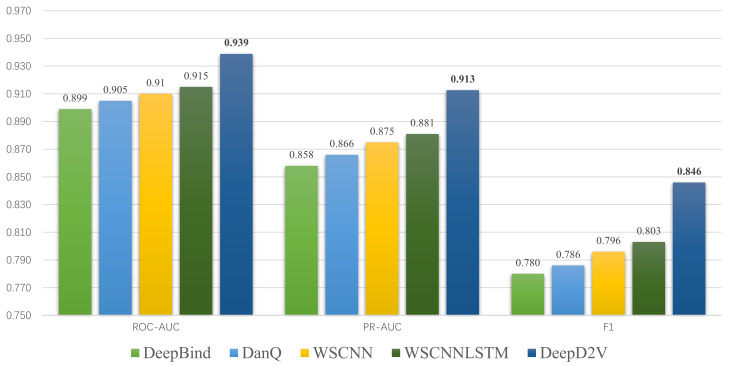
Comparison of DeepD2V and the competing methods on 50 ChIP-seq in-vivo data under the ROC AUC, PR AUC and F1-score metrics.

In summary, DeepD2V performs better than three other methods and achieves state-of-the-art performance on 50 in vivo ChIP-seq datasets on performance measure F1-score, PR AUC, and ROC AUC. The average score of the three metrics on 50 data sets is shown in Figure 9. Moreover, the ROC AUC values on different data sets are shown in Table 2. These results consistently indicate that DeepD2V performs superior to DeepBind, DanQ, and WSCNNLSTM.

## 4. Conclusions and Future Work

We developed a novel framework based on deep learning for protein–DNA binding prediction in this paper. First, the reverse complementary sequence has been taken into account to train the prediction model. We made a hypothesis that proteins may bind to the inverse sequence or complementary sequence rather than original sequence. Therefore, we consider various combinations of original, reverse, complementary, and reverse complementary sequences. Our experimental results verified that the combined sequence is remarkably beneficial for feature extraction. Second, the word embedding algorithm is employed in DeepD2V to train distributed representation of *k*-mers. The distributed representation improves the follow-up classification task and feature learning. In addition, we presented a new framework based on deep learning, which takes the combined DNA sequence as input and employs both CNN and bi-LSTM to extract high-order and long-term features. Compared with other deep learning methods based on sequence feature alone for protein–DNA binding prediction, DeepD2V achieved better robustness and performance.

Although DeepD2V achieves state-of-the-art performance, it still has limitations and we plan to improve it in the following aspects. First, DeepD2V can only be applied to sequences with the same length, but, in most cases, the lengths of sequences are unequal, which pose difficulty in the extraction of sequence features. Hence, the new algorithm will be introduced for the feature extraction of variable length sequences [39]. In addition, our proposed framework uses only DNA sequences, while we can actually take other types of features into account in our future work, such as PSSM [40] and customized k-mer statistics [41], which can be fused to improve the performance of our method.Moreover, the attention mechanism [42], which achieves great success in compute vision and natural language processing, may be also a promising way to identify the key bases underlying the protein–DNA interactions.

## Figures and Tables

**Table 1:** DeepD2V Hyper-parameters, search space, and recommendation.

Calibration Hyper-Parameters	Search Space	Recommendation
Convolutional layer number	{1, 3, 5, 7}	3
Learning rate	{$1\times 10^{-2}$, $1\times 10^{-3}$, $1\times 10^{-4}$, $1\times 10^{-5}$}	$1\times 10^{-3}$
Batch Size	{1, 32, 64, 128, 256, 512}	64
Loss Function	/	Binary cross entropy
Optimizer	{Adam, AdaDelta}	Adam
Convolutional neurons number	{8, 16, 32, 64, 128}	16
Convolutional kernel size	{3, 9, 16, 24}	3
Max Pooling window size	{2, 4, 8}	2
Number of bi-LSTM neurons	{8, 16, 32, 64}	16
Dropout ratio	{0.1, 0.2, 0.5}	0.1

**Table 2:** AUC performance metric of DeepD2V with other deep learning model.

Cell Line	TF	DeepBind	DanQ	WSCNNLSTM	DeepD2V	Cell Line	TF	DeepBind	DanQ	WSCNNLSTM	DeepD2V
Gm12878	Batf	0.887	0.904	0.906	0.939	H1hesc	Rad	0.973	0.978	0.981	0.984
Gm12878	Bcl1	0.823	0.831	0.844	0.907	H1hesc	Sin3	0.894	0.898	0.912	0.927
Gm12878	Bcl3	0.858	0.884	0.892	0.927	H1hesc	Sp1	0.874	0.874	0.885	0.930
Gm12878	Bclaf	0.843	0.852	0.866	0.898	H1hesc	Srf	0.949	0.956	0.964	0.966
Gm12878	Ebf	0.861	0.877	0.882	0.910	H1hesc	Taf1	0.908	0.911	0.912	0.927
Gm12878	Egr1	0.934	0.942	0.949	0.960	H1hesc	Tcf12	0.850	0.863	0.874	0.922
Gm12878	Elf1	0.905	0.914	0.912	0.927	H1hesc	Usf1	0.973	0.978	0.981	0.984
Gm12878	Ets1	0.912	0.868	0.929	0.951	H1hesc	Yy1	0.923	0.929	0.939	0.955
Gm12878	Irf4	0.833	0.815	0.847	0.891	K562	Atf3	0.931	0.945	0.952	0.954
Gm12878	Mef2a	0.852	0.837	0.876	0.927	K562	E2f6	0.935	0.943	0.945	0.958
Gm12878	Nrsf	0.899	0.905	0.915	0.937	K562	Egr1	0.947	0.954	0.960	0.967
Gm12878	Pax5c20	0.850	0.861	0.866	0.905	K562	Elf1	0.943	0.941	0.947	0.952
Gm12878	Pax5n19	0.845	0.845	0.872	0.928	K562	Ets1	0.0.883	0.893	0.891	0.918
Gm12878	Pbx3	0.855	0.879	0.879	0.931	K562	Fosl1	0.935	0.945	0.949	0.960
Gm12878	Pou2	0.819	0.835	0.843	0.894	K562	Gabp	0.932	0.927	0.943	0.961
Gm12878	Pu1	0.949	0.962	0.967	0.977	K562	Gata2	0.827	0.844	0.847	0.885
Gm12878	Rad21	0.978	0.985	0.988	0.988	K562	Hey1	0.875	0.879	0.875	0.891
Gm12878	Sp1	0.800	0.810	0.821	0.883	K562	Max	0.905	0.914	0.926	0.944
Gm12878	Srf	0.883	0.890	0.922	0.949	K562	Nrsf	0.880	0.883	0.901	0.943
Gm12878	Taf1	0.887	0.904	0.906	0.939	K562	Pu1	0.971	0.981	0.983	0.987
Gm12878	Tcf12	0.871	0.879	0.890	0.917	K562	Rad21	0.982	0.989	0.991	0.992
Gm12878	Usf1	0.918	0.948	0.953	0.961	K562	Srf	0.878	0.855	0.898	0.939
Gm12878	Yy1	0.888	0.891	0.901	0.927	K562	Taf1	0.898	0.909	0.909	0.925
H1hesc	Gabp	0.905	0.913	0.916	0.937	K562	Usf1	0.973	0.978	0.981	0.984
H1hesc	Nrsf	0.945	0.950	0.959	0.969	K562	Yy1	0.912	0.914	0.920	0.940
							Average ROC AUC	0.899	0.905	0.915	0.939

## Data Availability

The datasets analyzed during the current study are available in http://pre3sdn.denglab.org/rawdata.zip (accessed on 30 April 2021).

## Supplementary Materials

The following are available online at https://www.mdpi.com/article/10.3390/ijms22115521/s1, For detailed results of the experiments, please see the Supplementary Table S4.

## Abbreviations

CNN	Convolutional neural networks
RNN	Recurrent neural networks
LSTM	Long-term short-term memory networks
ROC	Receiver operating characteristic
PR	Precision recall
AUC	Area under the curve
