# Supplementary material for: DeepD2V: A Novel Deep Learning-Based Framework for Predicting Transcription Factor Binding Sites from Combined DNA Sequence
Source: Int J Mol Sci. 2021 May 24;22(11):5521. doi: 10.3390/ijms22115521 (PMC8197256; doi:10.3390/ijms22115521)
Supplement: Supplementary file 1 [file ijms-22-05521-s001.zip › ijms-1181522-SI.pdf]

Supplementary materials for DeepD2V: A Novel Deep Learning-Based Framework for Predicting Transcription Factor Binding Sites from Combined DNA Sequence

Supplementary Tables

Supplementary Table 1. Cross-validated ROC AUCs of various methods on 50 ChIP-seq datasets, and the best scores are highlighted in bold.

Cell Line	TF	Model 1 Deepbind	Model 2 DanQ	Model 3 WSCNNLSTM	DeepD2V	Cell Line	TF	Model 1 Deepbind	Model 2 DanQ	Model 3 WSCNNLSTM	DeepD2V
Gm12878	Batf	0.887	0.904	0.906	**0.939**	H1hesc	Rad	0.973	0.978	0.981	**0.984**
Gm12878	Bcl1	0.823	0.831	0.844	**0.907**	H1hesc	Sin3	0.894	0.898	0.912	**0.927**
Gm12878	Bcl3	0.858	0.884	0.892	**0.927**	H1hesc	Sp1	0.874	0.874	0.885	**0.930**
Gm12878	Bclaf	0.843	0.852	0.866	**0.898**	H1hesc	Srf	0.949	0.956	0.964	**0.966**
Gm12878	Ebf	0.861	0.877	0.882	**0.910**	H1hesc	Taf1	0.908	0.911	0.912	**0.927**
Gm12878	Egr1	0.934	0.942	0.949	**0.960**	H1hesc	Tcf12	0.850	0.863	0.874	**0.922**
Gm12878	Elf1	0.905	0.914	0.912	**0.927**	H1hesc	Usf1	0.970	0.978	0.983	**0.984**
Gm12878	Ets1	0.912	0.868	0.929	**0.951**	H1hesc	Yy1	0.923	0.929	0.939	**0.955**
Gm12878	Irf4	0.833	0.815	0.847	**0.891**	K562	Atf3	0.931	0.945	0.952	**0.954**
Gm12878	Mef2a	0.852	0.837	0.876	**0.927**	K562	E2f6	0.935	0.943	0.945	**0.958**
Gm12878	Nrsf	0.899	0.905	0.915	**0.937**	K562	Egr1	0.947	0.954	0.960	**0.967**
Gm12878	Pax5c20	0.850	0.861	0.866	**0.905**	K562	Elf1	0.943	0.941	0.947	**0.952**
Gm12878	Pax5n19	0.845	0.845	0.872	**0.928**	K562	Ets1	0.883	0.893	0.891	**0.918**
Gm12878	Pbx3	0.855	0.879	0.879	**0.931**	K562	Fosl1	0.935	0.945	0.949	**0.960**
Gm12878	Pou2	0.819	0.835	0.843	**0.894**	K562	Gabp	0.932	0.927	0.943	**0.961**
Gm12878	Pu1	0.949	0.962	0.967	**0.977**	K562	Gata2	0.827	0.844	0.847	**0.885**
Gm12878	Rad21	0.978	0.985	0.988	**0.988**	K562	Hey1	0.875	0.879	0.875	**0.891**
Gm12878	Sp1	0.800	0.810	0.821	**0.883**	K562	Max	0.905	0.914	0.926	**0.944**
Gm12878	Srf	0.883	0.890	0.922	**0.949**	K562	Nrsf	0.880	0.883	0.901	**0.943**
Gm12878	Taf1	0.897	0.905	0.901	**0.925**	K562	Pu1	0.971	0.981	0.983	**0.987**
Gm12878	Tcf12	0.871	0.879	0.890	**0.917**	K562	Rad21	0.982	0.989	0.991	**0.992**
Gm12878	Usf1	0.918	0.948	0.953	**0.961**	K562	Srf	0.878	0.855	0.898	**0.939**
Gm12878	Yy1	0.888	0.891	0.901	**0.927**	K562	Taf1	0.898	0.909	0.909	**0.925**
H1hesc	Gabp	0.905	0.913	0.916	**0.937**	K562	Usf1	0.947	0.955	0.961	**0.968**
H1hesc	Nrsf	0.945	0.950	0.959	**0.969**	K562	Yy1	0.912	0.914	0.920	**0.940**
						**Average ROC AUC**		0.899	0.905	0.915	**0.939**

Supplementary Table 2. Cross-validated PR AUCs of various methods on 50 ChIP-seq datasets, and the best scores are highlighted in bold.

Cell Line	TF	Model 1 Deepbind	Model 2 DanQ	Model 3 WSCNNLSTM	DeepD2V	Cell Line	TF	Model 1 Deepbind	Model 2 DanQ	Model 3 WSCNNLSTM	DeepD2V
Gm12878	Batf	0.799	0.832	0.833	**0.886**	H1hesc	Rad21	0.969	0.976	0.980	**0.982**
Gm12878	Bcl1	0.688	0.697	0.727	**0.824**	H1hesc	Sin3	0.885	0.890	0.898	**0.911**
Gm12878	Bcl3	0.767	0.810	0.821	**0.874**	H1hesc	Sp1	0.818	0.810	0.827	**0.893**
Gm12878	Bclaf	0.770	0.777	0.799	**0.847**	H1hesc	Srf	0.910	0.923	0.934	**0.938**
Gm12878	Ebf	0.823	0.847	0.850	**0.883**	H1hesc	Taf1	0.915	0.916	0.917	**0.927**
Gm12878	Egr1	0.928	0.936	0.940	**0.955**	H1hesc	Tcf12	0.717	0.734	0.759	**0.845**
Gm12878	Elf1	0.889	0.897	0.897	**0.911**	H1hesc	Usf1	0.948	0.962	0.966	**0.972**
Gm12878	Ets1	0.856	0.793	0.887	**0.916**	H1hesc	Yy1	0.905	0.913	0.923	**0.941**
Gm12878	Irf4	0.715	0.676	0.740	**0.811**	K562	Atf3	0.904	0.926	0.934	**0.937**
Gm12878	Mef2a	0.702	0.663	0.746	**0.850**	K562	E2f6	0.937	0.943	0.947	**0.956**
Gm12878	Nrsf	0.859	0.862	0.877	**0.909**	K562	Egr1	0.942	0.950	0.952	**0.964**
Gm12878	Pax5c20	0.857	0.867	0.879	**0.907**	K562	Elf1	0.960	0.955	0.961	**0.965**
Gm12878	Pax5n19	0.750	0.748	0.794	**0.871**	K562	Ets1	0.872	0.887	0.885	**0.902**
Gm12878	Pbx3	0.774	0.815	0.818	**0.881**	K562	Fosl1	0.894	0.905	0.915	**0.925**
Gm12878	Pou2	0.753	0.770	0.781	**0.838**	K562	Gabp	0.886	0.878	0.903	**0.935**
Gm12878	Pu1	0.911	0.939	0.942	**0.963**	K562	Gata2	0.730	0.768	0.771	**0.819**
Gm12878	Rad21	0.965	0.977	0.982	**0.982**	K562	Hey1	0.884	0.886	0.888	**0.897**
Gm12878	Sp1	0.671	0.696	0.714	**0.800**	K562	Max	0.916	0.923	0.933	**0.948**
Gm12878	Srf	0.793	0.800	0.850	**0.899**	K562	Nrsf	0.829	0.839	0.864	**0.917**
Gm12878	Taf1	0.863	0.878	0.878	**0.896**	K562	Pu1	0.959	0.975	0.980	**0.983**
Gm12878	Tcf12	0.850	0.860	0.874	**0.899**	K562	Rad21	0.974	0.984	0.984	**0.989**
Gm12878	Usf1	0.894	0.924	0.929	**0.941**	K562	Srf	0.807	0.764	0.832	**0.892**
Gm12878	Yy1	0.897	0.900	0.907	**0.932**	K562	Taf1	0.902	0.910	0.909	**0.921**
H1hesc	Gabp	0.919	0.923	0.931	**0.949**	K562	Usf1	0.919	0.933	0.935	**0.951**
H1hesc	Nrsf	0.910	0.919	0.932	**0.953**	K562	Yy1	0.924	0.924	0.931	**0.946**
						**Average PR AUC**		0.858	0.866	0.881	**0.913**

Supplementary Table 3. Cross-validated F1-scores of various methods on 50 ChIP-seq datasets, and the best scores are highlighted in bold.

Cell Line	TF	Model 1 Deepbind	Model 2 DanQ	Model 3 WSCNNLSTM	DeepD2V	Cell Line	TF	Model 1 Deepbind	Model 2 DanQ	Model 3 WSCNNLSTM	DeepD2V
Gm12878	Batf	0.719	0.734	0.747	**0.809**	H1hesc	Rad21	0.915	0.925	0.928	**0.942**
Gm12878	Bcl1	0.575	0.571	0.620	**0.748**	H1hesc	Sin3	0.814	0.822	0.841	**0.865**
Gm12878	Bcl3	0.666	0.695	0.728	**0.791**	H1hesc	Sp1	0.732	0.711	0.748	**0.814**
Gm12878	Bclaf	0.667	0.671	0.685	**0.762**	H1hesc	Srf	0.823	0.837	0.858	**0.872**
Gm12878	Ebf	0.724	0.744	0.754	**0.792**	H1hesc	Taf1	0.843	0.849	0.849	**0.872**
Gm12878	Egr1	0.865	0.878	0.882	**0.901**	H1hesc	Tcf12	0.655	0.671	0.682	**0.782**
Gm12878	Elf1	0.809	0.820	0.812	**0.841**	H1hesc	Usf1	0.894	0.908	0.915	**0.922**
Gm12878	Ets1	0.770	0.695	0.786	**0.840**	H1hesc	Yy1	0.825	0.838	0.846	**0.873**
Gm12878	Irf4	0.611	0.602	0.642	**0.734**	K562	Atf3	0.835	0.855	0.868	**0.866**
Gm12878	Mef2a	0.608	0.535	0.648	**0.773**	K562	E2f6	0.868	0.877	0.882	**0.905**
Gm12878	Nrsf	0.784	0.768	0.800	**0.838**	K562	Egr1	0.888	0.895	0.899	**0.914**
Gm12878	Pax5c20	0.770	0.783	0.782	**0.829**	K562	Elf1	0.916	0.913	0.922	**0.927**
Gm12878	Pax5n19	0.657	0.653	0.680	**0.799**	K562	Ets1	0.815	0.832	0.823	**0.846**
Gm12878	Pbx3	0.651	0.713	0.725	**0.807**	K562	Fosl1	0.856	0.861	0.859	**0.868**
Gm12878	Pou2	0.640	0.643	0.661	**0.761**	K562	Gabp	0.806	0.802	0.827	**0.861**
Gm12878	Pu1	0.852	0.884	0.890	**0.917**	K562	Gata2	0.637	0.673	0.668	0.733
Gm12878	Rad21	0.913	0.932	0.939	**0.946**	K562	Hey1	0.793	0.795	0.796	**0.810**
Gm12878	Sp1	0.541	0.574	0.568	**0.721**	K562	Max	0.846	0.855	0.865	**0.888**
Gm12878	Srf	0.686	0.703	0.749	**0.811**	K562	Nrsf	0.750	0.737	0.782	**0.834**
Gm12878	Taf1	0.798	0.813	0.806	**0.831**	K562	Pu1	0.907	0.929	0.932	**0.946**
Gm12878	Tcf12	0.759	0.767	0.785	**0.827**	K562	Rad21	0.922	0.941	0.940	**0.954**
Gm12878	Usf1	0.829	0.848	0.854	**0.866**	K562	Srf	0.717	0.664	0.741	**0.807**
Gm12878	Yy1	0.804	0.806	0.810	**0.847**	K562	Taf1	0.827	0.839	0.839	**0.861**
H1hesc	Gabp	0.864	0.870	0.877	**0.882**	K562	Usf1	0.854	0.866	0.867	**0.888**
H1hesc	Nrsf	0.837	0.846	0.862	**0.889**	K562	Yy1	0.852	0.854	0.864	**0.885**
						**Average F1-score**		0.780	0.786	0.803	**0.846**

Supplementary Table 4. We compare the performance of DeepD2V use one-hot encoding and DeepD2V use dna2vec respectively.

Supplementary Table 4.1: ROC AUC of DeepD2V on the 50 ChIP-seq datasets, and the best scores are highlighted in bold.

Cell Line	TF	DeepD2V		Cell Line	TF	DeepD2V	
		one-hot	dna2vec			one-hot	dna2vec
Gm12878	Batf	0.917	**0.939**	H1hesc	Rad	0.984	**0.984**
Gm12878	Bcl1	0.865	**0.907**	H1hesc	Sin3	0.913	**0.927**
Gm12878	Bcl3	0.910	**0.927**	H1hesc	Sp1	0.896	**0.930**
Gm12878	Bclaf	0.877	**0.898**	H1hesc	Srf	0.964	**0.966**
Gm12878	Ebf	0.889	**0.910**	H1hesc	Taf1	0.922	**0.927**
Gm12878	Egr1	0.938	**0.960**	H1hesc	Tcf12	0.889	**0.922**
Gm12878	Elf1	0.913	**0.927**	H1hesc	Usf1	0.980	**0.984**
Gm12878	Ets1	0.910	**0.951**	H1hesc	Yy1	0.933	**0.955**
Gm12878	Irf4	0.861	**0.891**	K562	Atf3	0.943	**0.954**
Gm12878	Mef2a	0.898	**0.927**	K562	E2f6	0.947	**0.958**
Gm12878	Nrsf	0.911	**0.937**	K562	Egr1	0.957	**0.967**
Gm12878	Pax5c20	0.879	**0.905**	K562	Elf1	0.945	**0.952**
Gm12878	Pax5n19	0.883	**0.928**	K562	Ets1	0.905	**0.918**
Gm12878	Pbx3	0.903	**0.931**	K562	Fosl1	0.952	**0.960**
Gm12878	Pou2	0.869	**0.894**	K562	Gabp	0.944	**0.961**
Gm12878	Pu1	0.966	**0.977**	K562	Gata2	0.866	**0.885**
Gm12878	Rad21	0.986	**0.988**	K562	Hey1	0.884	**0.891**
Gm12878	Sp1	0.830	**0.883**	K562	Max	0.924	**0.944**
Gm12878	Srf	0.911	**0.949**	K562	Nrsf	0.896	**0.943**
Gm12878	Taf1	0.919	**0.925**	K562	Pu1	0.984	**0.987**
Gm12878	Tcf12	0.894	**0.917**	K562	Rad21	0.989	**0.992**
Gm12878	Usf1	0.954	**0.961**	K562	Srf	0.909	**0.939**
Gm12878	Yy1	0.906	**0.927**	K562	Taf1	0.918	**0.925**
H1hesc	Gabp	0.924	**0.937**	K562	Usf1	0.959	**0.968**
H1hesc	Nrsf	0.961	**0.969**	K562	Yy1	0.918	**0.940**
				**Average ROC AUC**		0.920	**0.939**

Supplementary Table 4.2: PR AUC of DeepD2V on the 50 ChIP-seq datasets, and the best scores are highlighted in bold.

Cell Line	TF	DeepD2V		Cell Line	TF	DeepD2V	
		one-hot	dna2vec			one-hot	dna2vec
Gm12878	Batf	0.849	**0.886**	H1hesc	Rad	0.982	**0.982**
Gm12878	Bcl1	0.757	**0.824**	H1hesc	Sin3	0.901	**0.911**
Gm12878	Bcl3	0.846	**0.874**	H1hesc	Sp1	0.838	**0.893**
Gm12878	Bclaf	0.814	**0.847**	H1hesc	Srf	0.935	**0.938**
Gm12878	Ebf	0.860	**0.883**	H1hesc	Taf1	0.924	**0.927**
Gm12878	Egr1	0.928	**0.955**	H1hesc	Tcf12	0.785	**0.845**
Gm12878	Elf1	0.895	**0.911**	H1hesc	Usf1	0.964	**0.972**
Gm12878	Ets1	0.846	**0.916**	H1hesc	Yy1	0.914	**0.941**
Gm12878	Irf4	0.763	**0.811**	K562	Atf3	0.923	**0.937**
Gm12878	Mef2a	0.790	**0.850**	K562	E2f6	0.946	**0.956**
Gm12878	Nrsf	0.868	**0.909**	K562	Egr1	0.954	**0.964**
Gm12878	Pax5c20	0.883	**0.907**	K562	Elf1	0.961	**0.965**
Gm12878	Pax5n19	0.801	**0.871**	K562	Ets1	0.895	**0.902**
Gm12878	Pbx3	0.845	**0.881**	K562	Fosl1	0.914	**0.925**
Gm12878	Pou2	0.809	**0.838**	K562	Gabp	0.906	**0.935**
Gm12878	Pu1	0.945	**0.963**	K562	Gata2	0.792	**0.819**
Gm12878	Rad21	0.980	**0.982**	K562	Hey1	0.892	**0.897**
Gm12878	Sp1	0.719	**0.800**	K562	Max	0.933	**0.948**
Gm12878	Srf	0.834	**0.899**	K562	Nrsf	0.854	**0.917**
Gm12878	Taf1	0.887	**0.896**	K562	Pu1	0.978	**0.983**
Gm12878	Tcf12	0.876	**0.899**	K562	Rad21	0.985	**0.989**
Gm12878	Usf1	0.929	**0.941**	K562	Srf	0.843	**0.892**
Gm12878	Yy1	0.913	**0.932**	K562	Taf1	0.917	**0.921**
H1hesc	Gabp	0.936	**0.949**	K562	Usf1	0.937	**0.951**
H1hesc	Nrsf	0.935	**0.953**	K562	Yy1	0.929	**0.946**
				**Average ROC AUC**		0.886	**0.913**

Supplementary Table 4.3: F1-scores of DeepD2V on the 50 ChIP-seq datasets, and the best scores are highlighted in bold.

Cell Line	TF	DeepD2V		Cell Line	TF	DeepD2V	
		one-hot	dna2vec			one-hot	dna2vec
Gm12878	Batf	0.759	**0.809**	H1hesc	Rad	0.939	**0.942**
Gm12878	Bcl1	0.661	**0.748**	H1hesc	Sin3	0.826	**0.865**
Gm12878	Bcl3	0.749	**0.791**	H1hesc	Sp1	0.754	**0.814**
Gm12878	Bclaf	0.711	**0.762**	H1hesc	Srf	0.863	**0.872**
Gm12878	Ebf	0.751	**0.792**	H1hesc	Taf1	0.861	**0.872**
Gm12878	Egr1	0.858	**0.901**	H1hesc	Tcf12	0.729	**0.782**
Gm12878	Elf1	0.819	**0.841**	H1hesc	Usf1	0.912	**0.922**
Gm12878	Ets1	0.744	**0.840**	H1hesc	Yy1	0.840	**0.873**
Gm12878	Irf4	0.681	**0.734**	K562	Atf3	0.844	**0.866**
Gm12878	Mef2a	0.695	**0.773**	K562	E2f6	0.884	**0.905**
Gm12878	Nrsf	0.784	**0.838**	K562	Egr1	0.896	**0.914**
Gm12878	Pax5c20	0.794	**0.829**	K562	Elf1	0.909	**0.927**
Gm12878	Pax5n19	0.712	**0.799**	K562	Ets1	0.828	**0.846**
Gm12878	Pbx3	0.751	**0.807**	K562	Fosl1	0.854	**0.868**
Gm12878	Pou2	0.719	**0.761**	K562	Gabp	0.825	**0.861**
Gm12878	Pu1	0.893	**0.917**	K562	Gata2	0.708	**0.733**
Gm12878	Rad21	0.940	**0.946**	K562	Hey1	0.797	**0.810**
Gm12878	Sp1	0.620	**0.721**	K562	Max	0.868	**0.888**
Gm12878	Srf	0.736	**0.811**	K562	Nrsf	0.720	**0.834**
Gm12878	Taf1	0.789	**0.831**	K562	Pu1	0.935	**0.946**
Gm12878	Tcf12	0.786	**0.827**	K562	Rad21	0.944	**0.954**
Gm12878	Usf1	0.853	**0.866**	K562	Srf	0.744	**0.807**
Gm12878	Yy1	0.827	**0.847**	K562	Taf1	0.846	**0.861**
H1hesc	Gabp	0.879	**0.882**	K562	Usf1	0.872	**0.888**
H1hesc	Nrsf	0.868	**0.889**	K562	Yy1	0.861	**0.885**
				**Average ROC AUC**		0.809	**0.846**
